# Analysis of the Correlation between Commodity Grade and Quality of *Angelica sinensis* by Determination of Active Compounds Using Ultraperformance Liquid Chromatography Coupled with Chemometrics

**DOI:** 10.1155/2014/143286

**Published:** 2014-04-14

**Authors:** Zenghui Wang, Dongmei Wang, Linfang Huang

**Affiliations:** Institute of Medicinal Plant Development, Chinese Academy of Medical Sciences & Peking Union Medical College, Beijing 100193, China

## Abstract

The contents of ferulic acid, senkyunolide A, butylidenephthalide, ligustilide, and n-butylphthalide were determined by UPLC analytical method; the correlation among the grade, average weight, and content was explored by correlation analysis and analysis of variance (ANOVA); the different commercial grades with average weight and content were revealed by principal component analysis (PCA) and then rationality analysis grade classification of *A. sinensis*. The results showed that various commercial grades can be distinguished by PCA analysis. And there was significant negative correlation between the commodity grades and average weight, commodity, and the content of bioactive compounds, while the content of senkyunolide A had significant negative correlation with commodity grades (*P* < 0.01). Average weight had no correlation with chemicals compounds. Additionally, there was significant positive correlation among the bioactive compounds (content of ferulic acid and phthalides) of different grades of *A. sinensis*. The content of senkyunolide A, butylidenephthalide, and ligustilide had significant positive correlation with the content of ferulic acid. The content of ligustilide and butylidenephthalide had significant positive correlation with the content of senkyunolide A. The content of ligustilide had significant positive correlation with the content of butylidenephthalide. The basis of grades classification is related with the difference levels of the bioactive compounds.

## 1. Introduction

Commercial specifications and grades of Chinese medicinal materials, the summary of practical experience (such as shape, size, texture, color, odor, and taste are based primarily on the human senses) for quality evaluation of traditional Chinese medicine, were the reflect of the quality of traditional Chinese medicine, which had played an important role in market circulation and ensuring clinical safety because it is easy, fast, and effective. However, most of these evaluation methods were still in the stage of experiential description which made it difficult to be inherited and applied. So far, the SFDA (State Food and Drug Administration) only issued 3 times of the commercial specification, involving 76 varieties, compared with the amount of commonly used trading of medicinal materials (more than 600 kinds); it is difficulty to satisfy the market demand [1].

*Angelica sinensis *(Danggui in Chinese) is one of the main exports of traditional Chinese medicine, derived from root of* Angelica sinensis* (Oliv.) Diels (Umbelliferae) [2], a well-known Chinese herbal medicine, first documented in Shennong Bencao Jing (Shennong's Classic of Materia Medica; 200–300 AD) [3, 4]. It has been used historically as a tonic, hematopoietic, and anti-inflammatory agent for the treatment of gynecological diseases such as menstrual disorders, amenorrhea, and dysmenorrheal for thousands of years in traditional Chinese Medicinal prescriptions [5, 6]. It has also been widely marketed as health food for women's care in Asia [7] and as a dietary supplement in Europe and America [8, 9]. However, quality assessment in the markets is extremely difficult and impractical when considering large number of export of this product.

Therefore, it is necessary to investigate the grades and quality assessment of* A. sinensis* [10–12]. This research took* A. sinensis* as the study subject. We collected different commodity grades of* A. sinensis* in the main producing areas and determined the content of ferulic acid and ligustilides, which are thought to be the biologically active components [13–17] by UPLC method for studies of the correlation between grades and quality. The results are helpful to provide the basis for the establishment of medicinal industry standards.

## 2. Materials and Methods

### 2.1. Plant Materials

**Figure 1:**
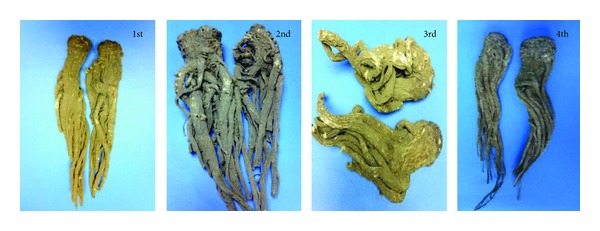
Different commercial grades of* A. sinensis* samples.

All the samples of* A. sinensis *were collected from Gansu, Yunnan, Sichuan, and Hubei province of China (Table 1, Figure 1). All the herbal samples were authenticated by Professor Lin Yulin, and the voucher specimens were deposited in the Institute of Medicinal Plant Development, Chinese Academy of Medical Sciences, Beijing, China.

### 2.2. Chemicals and Reagents

UPLC grade acetonitrile was from ThermoFisher, USA, analytical grade formic acid from Fisher, USA, and analytical grade method from Beijing chemical factory and deionized water was obtained from a Milli-Q water system (Millipore, Bedford, MA, USA). Ligustilide (Batch number: MUST-11072416), n-butylphthalide (Batch number: MUST-12020706), butylidenephthalide (Batch number: MUST-12071103), and senkyunolide A (Batch number: MUST-10102309) were purchased from the Chengdu Mansite, Biological Technology Co., Ltd. (all with purities > 98%).

### 2.3. Instrumentation

Instrumentation included Waters Acuity UPLC-PDA (Waters, USA, including quaternary solvent delivery system, vacuum degasser, autosampler, and Empower2 chromatography workstation), electronic analytical balance (Mettler, AB135-S), and Electro-Thermostatic Water Bath (Beijing Analytical Instrument Factory).

### 2.4. Preparation of Standard and Sample Solutions

The five reference compounds were accurately weighed: 1 mg dissolved in 10 mL volumetric flask with 70% methanol to produce standard stock solutions. The stock solution was diluted to yield a series of standard solution in the concentration range of 32–1722 *μ*g/mL, 30–415 *μ*g/mL, 156–722 *μ*g/mL, 332–6786 *μ*g/mL, and 33–2543 *μ*g/mL for ferulic acid, senkyunolide, n-butylphthalide, ligustilide, and butylidenephthalide, respectively. Samples of herbal materials were ground into fine powder then passed through a 20 mesh (0.9 mm) sieve. Sample powder (0.2 g) was accurately weighed and transferred into a 60 mL round bottom flask. 70% methanol (20 mL) was added and refluxed for 30 min. When cool, the methanol was added to compensate for weight loss. After filtering through a 0.22 *μ*m filter membrane, the filtrate was ready to be used.

### 2.5. Ultraperformance Liquid Chromatography

A 2 *μ*L aliquot was analyzed on a 2.1 × 100 mm ACQUITY 1.7 *μ*m BEH C_18_ column (Waters, Milford, MA) and maintained at 35°C using an ACQUITY UPLC system (Waters, Milford, MA). The mobile phases consisted of (a) acetonitrile and (b) water containing 0.1% formic acid. The UPLC elution conditions were optimized as follows: 95% A held for (2–4 min), 95% to 76% A (4–7 min), and 76% to 72% A (7-8 min), followed by 72% to 50% A (8–10 min), 50% to 30% A (10–12 min), 30% to 0% A (12–14 min), 0% A (held for 14-15 min), and 0% to 95% A (15-16 min). The flow rate was set at 0.30 mL/min. The column and autosampler were maintained at 35 and 5°C, respectively. The scan range for PDA was 261 nm and 281 nm. All experiments were performed in triplicate.

### 2.6. Validation of the UPLC Method

#### 2.6.1. Calibration Curves

Methanol stock solutions of 5 reference compounds were prepared and diluted to appropriate concentrations for the construction of calibration curves. Six concentrations of the solution were analysed in triplicate; the calibration curves were constructed by plotting the peak areas against the concentrations of the analytes. The stock solutions mentioned above were diluted to a series of appropriate concentrations with methanol, and an aliquot of the diluted solutions were injected into UPLC-DAD for analysis. The stock solutions mentioned above were diluted to a series of appropriate concentrations with methanol, and an aliquot of the diluted solutions were injected into UPLC-DAD for analysis. The limits of determinations (LODs) and limits of quantifications (LOQs) under the present conditions were determined at signal to noise (S/N) ratio of about 3 and 10, respectively.

#### 2.6.2. Precision, Accuracy, and Stability

Intra- and interday variations were chosen to determine the precision of the UPLC method. 0.2 g of* A. sinensis* sample was extracted and analysed as described in Sections 2.3 to 2.5. The intraday variability test was performed by triplicate extraction and analysed during a single day. The interday variability test was carried out on three different days. Variations were expressed by the relative standard deviations (R.S.D.) for intra- and interday. The recovery test was used to evaluate the accuracy of the method. Accurate amounts of ferulic acid, senkyunolide, n-butylphthalide, ligustilide, and butylidenephthalide were added to approximately 0.25 g of* A. sinensis* sample and then extracted and analysed as described in Section 2.3. The average recoveries were calculated by the following formula: recovery(%) = (amount found − regional amount)/amount spiked × 100%. For the stability test,* A. sinensis* sample was accurately weighed (approximately 0.5 g) and ultrasonic-extracted with 2.0 mL of methanol for 30 min at room temperature. The samples were then analysed at 0, 2, 4, 6, 8, 12, 24, and 36 h with the established method. The relative standard deviations (R.S.D.) of five data were used to evaluate the stability.

### 2.7. Statistical Methods

Spearman rank correlation analysis was carried out by SPSS system for windows release version 19.0 (SPSS Institute, Cary, NC, USA), the normality, mean, standard deviation (SD), median, and the outlier range of the* A. sinensis*; the values of the standard quality characteristics were acquired on the basis of mean ± SD.

## 3. Results and Discussion

### 3.1. Method Optimization

In sample preparation procedure, multifarious solvents, such as different concentrations (10%, 30%, 50%, 70%, and 90%) of ethanol and methanol, were tested and 70% methanol solution was selected because of its excellent dissolving capacity for* A. sinensis* sample. For UPLC analysis, two mobile phase systems, including acetonitrile-water and methanol-water, in various proportions were compared and different mobile phase additives, such as phosphate buffer, formic acid, and acetic acid, were also investigated, and finally, 0.1% formic acid aqueous solution and acetonitrile were used as mobile phases which could provide satisfactory separation and peak shapes of investigated compounds. In addition, ferulic acid and phthalides substances absorb different wavelengths under the UV conditions. Ten times difference of response value in the same substance under different wavelengths. 281 nm was employed as the detection wavelength since the difference of response value among ferulic acid, senkyunolide A, n-butylphthalide, and ligustilide compared with the response values under the maximum absorption wavelength was not obvious, while response values under 281 nm are only 1/10 compared with 261 nm, which is difficult to observe in the chromatogram. Therefore, 261 nm was chosen as detection of butylidenephthalide wavelength.

### 3.2. Method Validation

The linearity, regression, and linear ranges of five analytes were determined using the developed UPLC-PDA method. The data indicated a good relationship between concentrations and peak areas of the analytes within the test ranges (*R* ^2^ ≥ 0.9990). The LOQs and LODs of all analytes were less than 4.15 and 11.06 mg/mL. The overall RSDs of intra- and interday variations for analytes were not more than 4.49% and 6.54%, respectively. The established method also had acceptable accuracy with spike recovery of 98.33–102.22% for all analytes. As to stability test, the RSDs of the peak areas for analytes detected within 24 h were lower than 4.72% (Table 2). All these results demonstrated that the developed UPLC method was linear, sensitive, precise, accurate, and stable enough for simultaneous quantification of the five investigated compounds in* A. sinensis*.

### 3.3. Quality of Five Analytes in* A. sinensis* Samples

**Figure 2:**
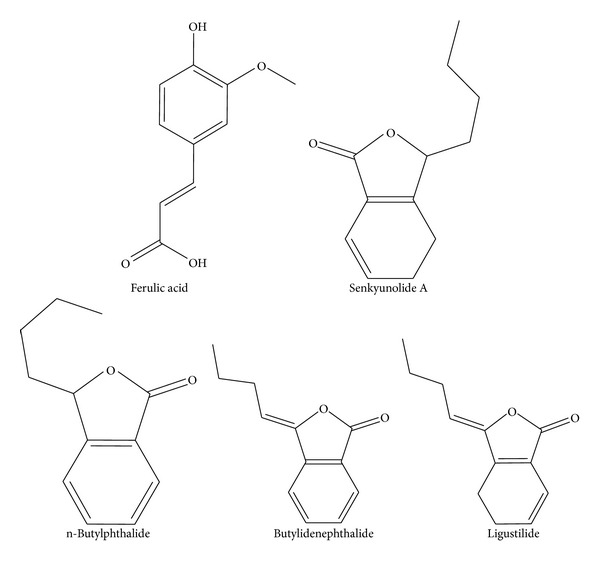
Chemical structures of ferulic acid and phthalides.

**Figure 3:**
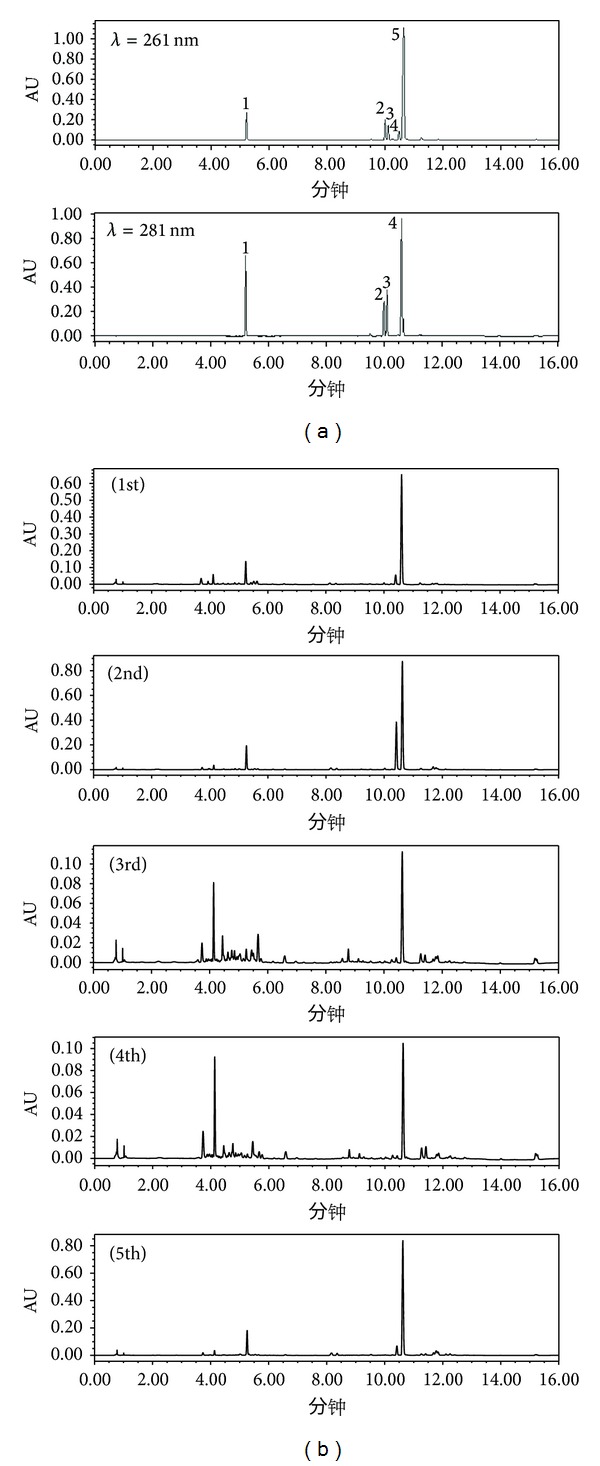
The UPLC chromatograms of reference standards (a) and different commercial grades* A. sinensis* samples (b). (1) Ferulic acid. (2) Senkyunolide A. (3) n-Butylphthalide. (4) Ligustilide. (5) Butylidenephthalide.

The developed UPLC method was successfully employed for simultaneous determination of the five major active components in 19* A. sinensis* samples collected from different localities. Typical chromatograms of reference compounds (a) and* A. sinensis* samples (b) were shown in Figure 3. The chemical structure of the reference compounds were shown in Figure 2. The identification of the investigated compounds was carried out by comparison of their retention time and UV spectra with reference chemicals. The contents of five investigated compounds in 19* A. sinensis* samples were summarized in Table 3.

**Figure 4:**
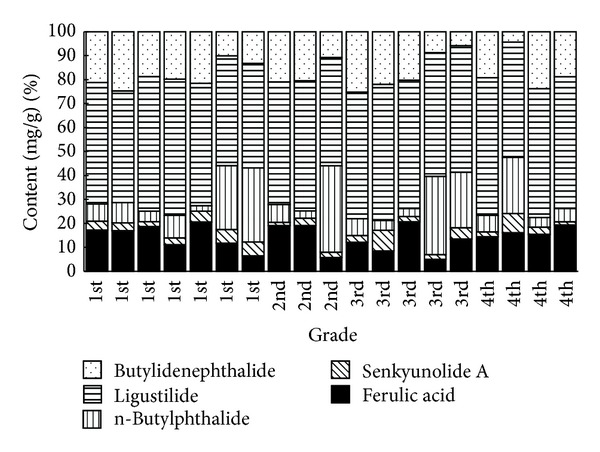
The content of ferulic acid, senkyunolide A, n-butylphthalide, ligustilide, and butylidenephthalide for different commercial grades of* A. sinensis* samples.

19 batches of different region of* A. sinensis* were determined, as shown in Figure 4. The sequence of ferulic acid content is Yunnan (0.14%~0.15%) > Gansu (0.04%~0.12%) > Sichuan (0.01%~0.02%) > Hubei (0.005%~0.006%). The ferulic acid contents in Yunnan and Gansu were accorded with the quality level request in Chinese Pharmacopoeia 2010 (≥0.05%), of which the highest content of ferulic acid was Yunnan sample. While the ferulic acid content of* A. sinensis* in Sichuan and Hubei was far below the standard of Chinese Pharmacopeias, they were substandard samples.

### 3.4. The Correlation among the Main Chemical Compounds, Average Weight, and Grades

The grade of whole* A. sinensis* was divided into five by using grading measurement method. From the data on the correlation coefficients between the main chemical compounds, average weight versus grades in Table 4, respectively, we can see that significantly negative correlation exists between average dry weight versus grades and the content of senkyunolide A versus grades (*P* < 0.01), which indicates that the higher the grade of* A. sinensis*, the heavier the weight. From the level of 1, 2, 3, 4, to 5, the average weight decreased gradually; the result coincident with the fact. And the content of senkyunolide A decreased with the increase of grade. Average weight had no correlation with the main chemicals compounds.

**Figure 5:**
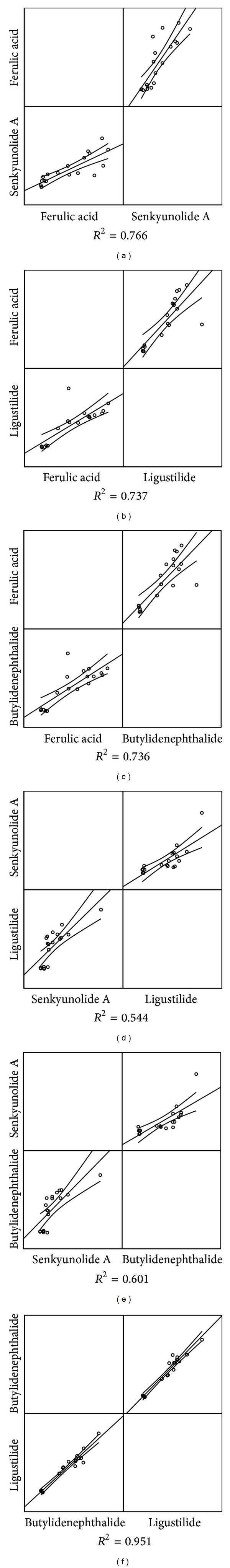
The correlation among chemical ingredients of* A. sinensis.*

Table 5 showed that 1–5 grades were analyzed by ANOVA; there were significant differences in content of ferulic acid and phthalides of different grades of* A. sinensis* (Table 5). As shown in Figure 5, the content of senkyunolide A, butylidenephthalide, and ligustilide had significant positive correlation with the content of ferulic acid. The content of ligustilide and butylidenephthalide had significant positive correlation with the content of senkyunolide A. The content of ligustilide had significant positive correlation with the content of butylidenephthalide. We found the content of ligustilide somehow related to the content of ferulic acid, senkyunolide A, and butylidenephthalide, which indicates that ligustilide and senkyunolide A serious possibility be one of the pharmacological biomarkers compounds in* A. sinensis* [18–22].

**Figure 6:**
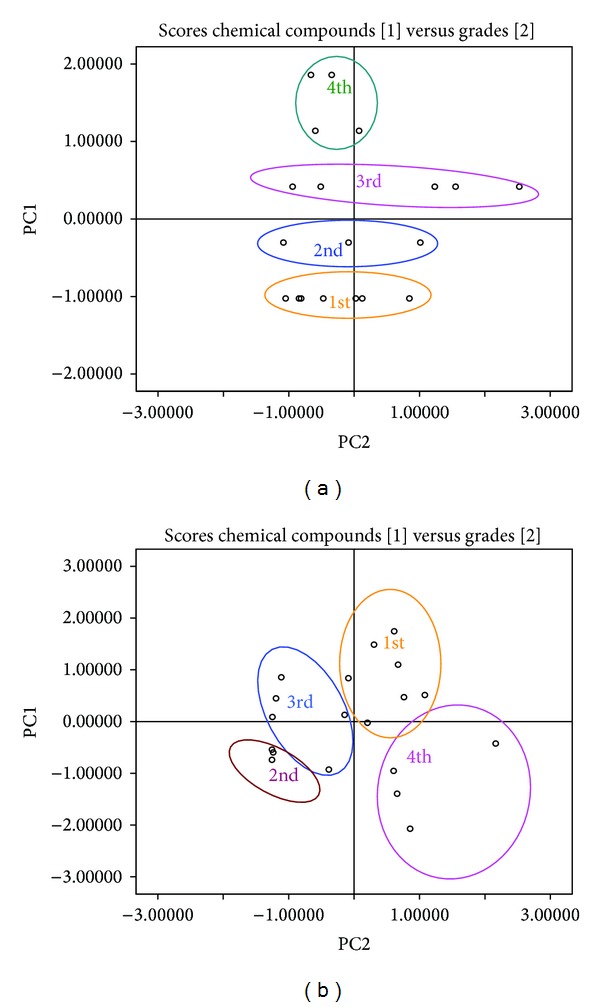
PCA (scores plot) of average weight versus grades (a) and chemical compounds versus grades (b) of* A. sinensis.*

Figure 6 shows that* A. sinensis* samples were divided into 5 main clusters observed in the PCA scores plot. In Figure 6(a), such division indicated that different grades could significantly distinguish from 1st to 5th by the different average weights of* A. sinensis*, while Figure 6(b) indicated that grades were distinctly separated by different contents of the chemical compounds. This result reflected rationality of grades classification.

## 4. Conclusion

In summary, the contents of ferulic acid, senkyunolide A, butylidenephthalide, ligustilide, and n-butylphthalide were determined by UPLC analytical method. The correlation among the grade, average weight, and content was explored by correlation analysis and analysis of variance (ANOVA); the different commercial grades with average weight and content were revealed by principal component analysis (PCA) and then rationality analysis classification of grade and quality of* A. sinensis*. The results showed that various commercial grades can be distinguished by PCA analysis. And there were significant negative correlation between the commodity grades and average weight, commodity, and the content of bioactive compounds. While the content of senkyunolide A had significant negative correlation with commodity grades (*P* < 0.01), average weight had no correlation with chemicals compounds. Additionally, there was significant positive correlation among the bioactive compounds (content of ferulic acid and phthalides) of different grades of* A. sinensis*. The content of senkyunolide A, ligustilide, and butylidenephthalide had significant positive correlation with the content of ferulic acid. The content of ligustilide and butylidenephthalide had significant positive correlation with the content of senkyunolide A. The content of ligustilide had significant positive correlation with the content of butylidenephthalide.

Quality standards for* A. sinensis* components are described in the Chinese Pharmacopoeia, such as percentage of total ash, acid-insoluble ash, and alcoholic extract content; ferulic acid content is also described. Our results suggest that senkyunolide A is a candidate for an efficient marker to define a radix quality standard. This work will provide useful references for the quantification and standardization of quality control for* A. sinensis*.

## Supplementary Material

The relationship of the average weight, commercial grades and chemical compounds.

## Figures and Tables

**Table 1:** *A. sinensis* samples used in this study.

Commercial grades	Source	Species	Collection year
1st	Minxian, Gansu Cultivated in the normal soil for two years	*A. sinensis *	2012
1st	Minxian, Gansu Cultivated in the normal soil for two years	*A. sinensis *	2012
1st	Weiyuan, Gansu Cultivated in the normal soil for two years	*A. sinensis *	2012
1st	Minxian, Gansu Cultivated in the normal soil for two years	*A. sinensis *	2012
1st	Heqing, Yunnan Cultivated in the normal soil for two years	*A. sinensis *	2012
1st	Deyang, Sichuan Cultivated in the normal soil for two years	*A. sinensis *	2012
1st	Deyang, Sichuan Cultivated in the normal soil for two years	*A. sinensis *	2012
2nd	Dangxian, Gansu Cultivated in the normal soil for two years	*A. sinensis *	2012
2nd	Heqing, Yunnan Cultivated in the normal soil for two years	*A. sinensis *	2012
2nd	Shiyan, Hubei Cultivated in the normal soil for two years	*A. sinensis *	2012
3rd	Minxian, Gansu Cultivated in the normal soil for two years	*A. sinensis *	2012
3rd	Weiyuan, Gansu Cultivated in the normal soil for two years	*A. sinensis *	2012
3rd	Heqing, Yunnan Cultivated in the normal soil for two years	*A. sinensis *	2012
3rd	Deyang, Sichuan Cultivated in the normal soil for two years	*A. sinensis *	2012
3rd	Shiyan, Hubei Cultivated in the normal soil for two years	*A. sinensis *	2012
4th	Weiyuan, Gansu Cultivated in the normal soil for two years	*A. sinensis *	2012
4th	Deyang, Sichuan Cultivated in the normal soil for two years	*A. sinensis *	2012
4th	Weiyuan, Gansu Cultivated in the normal soil for two years	*A. sinensis *	2012
4th	Weiyuan, Gansu Cultivated in the normal soil for two years	*A. sinensis *	2012

**Table 2:** Calibration curves, LODs, LOQs, and precision for ferulic acid, senkyunolide A, n-butylphthalide, ligustilide, and butylidenephthalide.

Reference samples	Calibration curves	*R* ^2^	Test ranges (mg/mL)	LODs (*μ*g/mL)	LOQs (*μ*g/mL)
Ferulic acid	*Y* = 3*E* + 07*X* + 2835.3	0.9999	0.032~1.722	4.15	11.06
Senkyunolide A	*Y* = 2*E* + 07*X* + 5256.2	1	0.030~0.415	4.57	11.78
n-Butylphthalide	*Y* = 7*E* + 07*X* + 295390	0.999	0.156~0.722	5.33	12.56
Ligustilide	*Y* = 6*E* + 07*X* + 11594	0.9999	0.332~6.786	5.74	13.32
Butylidenephthalide	*Y* = 9*E* + 07*X* + 420771	0.9991	0.033~2.543	5.12	12.73

**Table 3:** Contents of ferulic acid, phthalides, and average weight of *A*.  *sinensis* for different commercial grades.

Grade	Ferulic acid mg/g	Senkyunolide A mg/g	n-Butylphthalide mg/g	Ligustilide mg/g	Butylidenephthalide mg/g	Average weight g
1st	1.061	0.226	0.442	3.121	1.306	28.429
1st	1.094	0.215	0.551	3.002	1.589	42.592
1st	0.842	0.099	0.194	2.540	0.847	34.265
1st	0.405	0.101	0.340	2.060	0.717	33.457
1st	1.364	0.301	0.153	3.379	1.431	54.81
1st	0.113	0.055	0.255	0.440	0.097	57.253
1st	0.059	0.054	0.284	0.400	0.121	74.985
2nd	1.198	0.086	0.468	3.215	1.309	27.598
2nd	1.496	0.235	0.244	4.243	1.603	79.09
2nd	0.056	0.020	0.343	0.431	0.103	52.095
3rd	0.622	0.141	0.365	2.695	1.295	41.63
3rd	0.836	0.829	0.411	5.551	2.146	31.045
3rd	1.389	0.142	0.226	3.582	1.362	116.31
3rd	0.15	0.052	0.258	0.588	0.065	84.52
3rd	0.049	0.019	0.313	0.497	0.083	92.35
4th	0.653	0.090	0.311	2.596	0.865	39.605
4th	0.185	0.092	0.27	0.552	0.051	56.053
4th	0.978	0.189	0.258	3.396	1.509	45.748
4th	1.085	0.077	0.310	3.081	1.053	37.943

**Table 4:** The correlation among the main chemical compounds, weight, and grades of *A. sinensis*.

	Grade	Average weight	Ferulic acid	Senkyunolide	n-Butylphthalide	Ligustilide	Butylidenephthalide
Grade	1.000						
Average weight	−0.603**	1.000					
Ferulic acid	−0.089	0.01	1.000				
Senkyunolide A	−0.501*	−0.031	0.682**	1.000			
n-Butylphthalide	0.019	−0.361	−0.143	0.015	1.000		
Ligustilide	0.088	−0.012	0.863**	0.568*	0.903	1.000	
Butylidenephthalide	−0.029	−0.022	0.805**	0.582**	0.616	0.895**	1.000

**P < 0.01 (bilateral). The correlation is significant.

*P < 0.05. The correlation is significant.

**Table 5:** Variance analysis of average weight, the chemical compounds, and grades.

		AVG	DF	SD	SE	*P*
Grade	Average weight	51.7778	18	24.5124	5.6235	0.046
	Ferulic acid	−1.7034	18	1.4675	0.3367	0.94
	Senkyunolide A	−2.2619	18	1.3978	0.3209	0.985
	n-Butylphthalide	−2.1055	18	1.4026	0.3218	0.614
	Ligustilide	−0.03321	18	1.9209	0.4406	0.619
	Butylidenephthalide	−1.4973	18	1.5016	0.3445	0.811

Average weight	Ferulic acid	53.4812	18	24.6503	5.6551	0.621
	Senkyunolide A	54.0397	18	24.6324	5.6511	0.271
	n-Butylphthalide	53.8832	18	24.6305	5.6507	0.042
	Ligustilide	51.8110	18	25.0694	5.7513	0.221
	Butylidenephthalide	53.2751	18	24.8041	5.6904	0.181

Ferulic acid	Senkyunolide A	0.5585	18	0.4661	0.1069	0.010
	Ligustilide	−1.6702	18	1.1058	0.2537	0.000
	Butylidenephthalide	−0.2061	18	0.3458	0.0793	0.000

Senkyunolide A	Ligustilide	−2.2287	18	1.3804	0.3167	0.000
	Butylidenephthalide	−0.7647	18	0.5475	0.1256	0.001

Ligustilide	Butylidenephthalide	1.4641	18	0.8750	0.2007	0.000

*P* < 0.05, significant difference.
