# Supplementary material for: Analysis of the Correlation between Commodity Grade and Quality of Angelica sinensis by Determination of Active Compounds Using Ultraperformance Liquid Chromatography Coupled with Chemometrics
Source: Evid Based Complement Alternat Med. 2014 Apr 14;2014:143286. doi: 10.1155/2014/143286 (PMC4009318; doi:10.1155/2014/143286)
Supplement: Supplementary file 1 — The relationship of the average weight, commercial grades and chemical compounds. [file 143286.f1.doc]

**Negative correlation**

**Average**

**weight**

**Commercial grades**


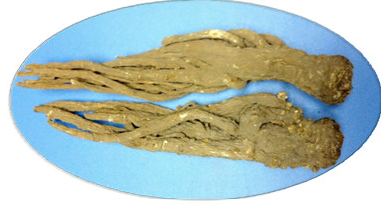


***Angelica sinensis* (Danggui in Chinese)**

**Chemical**

**compounds**

**Positive**

**Ferulic**

**acid**

**Phthalides**

**correlation**
